# Influence of nonylphenol exposure on basic growth, development, and thyroid tissue structure in F1 male rats

**DOI:** 10.7717/peerj.7039

**Published:** 2019-06-17

**Authors:** Lin Wang, Jie Xu, Feng Zeng, Xiangjun Fu, Weihong Xu, Jie Yu

**Affiliations:** 1 School of Public Health, Zunyi Medical University, Zunyi, China; 2 Breast & Thyroid Disease Medical Center, Affiliated Hospital of Zunyi Medical University, Zunyi, China

**Keywords:** Gestational and lactational, Nonylphenol, Thyroid, Ultrastructure, F1 male rats

## Abstract

**Objective:**

Environmental endocrine disruptors (EEDs) with a weak ability to mimic estrogen have been associated with thyroid dysfunction. However, little is known about the effect of nonylphenol (NP), a well-known EED, on thyroid structure. The present study evaluates whether gestational and lactational exposure to NP impacts growth and thyroid structure in F1 male rats.

**Methods:**

A total of 60 rats were gavaged with NP (25, 50, and 100 mg/kg), estradiol (E_2_, 30 μg/kg/day), and corn oil alone (vehicle control) from gestational day 6 to postnatal day (PND) 21. Serum thyroid hormones free triiodothyronine (FT3), free thyroxine (FT4) and thyroid stimulating hormone levels were detected by automated chemiluminescence immunoassay analyzer. The NP level in the thyroid was measured using high-performance liquid chromatography. The ultrastructure of follicular epithelial cells was examined using transmission electron microscopy. Histopathology was conducted using hematoxylin and eosin staining.

**Results:**

On PND 0, exposure to 50 and 100 mg/kg/day NP led to a significant decrease in the average litter size, litter weight and number of live pups per litter compared to the control group (*P* < 0.05). Dams exposed to NP during perinatal period demonstrated decreased serum levels of FT3 and FT4 in F1 male rats, when compared to the control group (*P* < 0.05). The NP level in the control group was 3.39 ± 0.08 ng/mg, while NP levels in the low, middle, and high dose groups ranged from 5.20 to 11.00 ng/mg. Exposure caused a dose-related increase in NP level in the thyroid of male pups (*P* < 0.01). The thicknesses of the thyroid follicular epithelium were 2.06 ± 0.37 μm in the control group and 3.97 ± 1.61 μm in the high-dose group. The thickness of the thyroid follicular epithelium increased with an increase in treatment dose in a dose-dependent manner (*P* < 0.05). The sizes of the thyroid follicles were 1,405.53 ± 866.62 μm^2^ in the control group and 317.49 ± 231.15 μm^2^ in the high-dose group. With increasing NP dosages, animals showed a decreased size of the thyroid follicle (*P* < 0.01). Thyroid follicular cells of NP-treated rats showed mildly swollen mitochondria and dilated rough endoplasmic reticulum in the cytoplasm.

**Conclusion:**

Nonylphenol can cross the placental barrier and accumulate in the thyroid of F1 male rats. Gestational and lactational exposure to NP in dams impacted both development and growth of pups and damaged the ultrastructure of their thyroid tissue, which may further negatively influence normal thyroid function.

## Introduction

Environmental endocrine disruptors (EEDs) are a research hotspot in environmental hygiene. Many investigations reported that EEDs can damage multiple systems (such as endocrine, reproductive, and immune systems), which negatively influences people’s health (Ponzo & Silvia, 2013; Tingi et al., 2016). Nonylphenol (NP) is a representative type of EED. It widely exists in the environment at relatively high levels (Yu et al., 2017). NP has been reported to damage endocrine function by simulating the effect of estrogen (Couderc et al., 2014), and can damage both tissues and organs in neonatal rats by overcoming the placental barrier and via breastfeeding (Chang, Wun & Wang, 2012; Ponzo & Silvia, 2013). It was also detected in human serum (Luo et al., 2016), breast milk (Sengul & Cevdet, 2017), urine (Yu et al., 2016a), adipose tissue (Yu et al., 2017), and semen (Rehman et al., 2018). Table 1 lists the levels of NP that were detected in the organs or tissues of animals. Growing evidence demonstrated that an increasing risk of hepatic (Yu et al., 2016b), renal (Yen et al., 2012), splenic (Hung et al., 2013), cardiac (Gao et al., 2015), and cerebral (Kazemi et al., 2018) diseases in animals and humans were associated with increased NP exposure. Furthermore, many of studies reported that EEDs (i.e., NP) could not only influence the exposed individuals, but also their offspring. However, the potential impacts of gestational and lactational exposure to NP on the thyroid structure have not been reported to date.

**Table 1:** Levels of NP detected in the organs or tissues of animals.

Tissue	NP concentration	Exposure dosage	Gavage period	Reference
Liver, rat	9.015–19.953 μg/mL	50, 100, 200 mg/kg	15 days	Luo et al. (2015)
Liver, rat	0.061–3.308 μg/g	50, 100, 200 mg/kg	3 days	Xiao, Li & Wu (2004)
Kidney, rat	0.031–2.723 μg/g	50, 100, 200 mg/kg	3 days	Xiao, Li & Wu (2004)
Heart, rat	0.022–0.285 μg/g	50, 100, 200 mg/kg	3 days	Xiao, Li & Wu (2004)
Brain, rat	0.029–0.305 μg/g	50, 100, 200 mg/kg	3 days	Xiao, Li & Wu (2004)
Testis, rat	0.71–5.48 μg/mL	5, 25, 125 μg/kg	35 days	Kazemi et al. (2016)
Pancreas, rat	2,045.0 ± 130.1 μg/L	200 mg/kg	60 days	Yang et al. (2017)
Serum, F1 rat	2.48 ± 0.32 ng/mL	25 μg/kg	15 days (F0)	Zhang et al. (2018)
Brain, mouse	4.94–5.16 ng/g	–	–	Sun et al. (2016)
Heart, mouse	16.28 ng/g	–	–	Sun et al. (2016)
Liver, mouse	2.24–2.94 ng/g	–	–	Sun et al. (2016)
Subcutaneous adipose tissue, human	9.8–266.5 ng/g	–	–	Ferrara et al. (2011)
Cyprinus carp meat	1.92 ± 0.33	–	–	Mortazavi et al. (2013)
Liver of cyprinus carp	3.21 ± 0.46	–	–	Mortazavi et al. (2013)
Fish meat	20–100 ng/g	–	–	Shao et al. (2005)
Crucian carp meat	0.5–15 ng/g	–	–	Liu et al. (2011)
Nile tilapia meat	0.031–0.053 μg/g	500 μg/L	10 days	Ismail & Mahboub (2016)
Liver of red mullet	33.7 ± 9.9 ng/g	–	–	Errico et al. (2017)
Red mullet meat	18.6 ± 3.5 ng/g	–	–	Errico et al. (2017)

The incidence of thyroid diseases is continuously increasing (Tingi et al., 2016; Bajaj, Salwan & Salwan, 2016). The worldwide incidence has been reported to increase from 1.6/100,000 in 1990 to 3.2/100,000 in 2016 (Li & Li, 2018). The average detection rate of thyroid nodules in China increased from 29.8% in 2008 to 41.3% in 2014 (Wang et al., 2018). The incidence of thyroid diseases is related to exposure to EEDs (Wetherill et al., 2007; Benedetti et al., 2017). Bisphenol A (BPA), poly brominated diphenyl ethers, and polychlorinated biphenyl (PCBs) exert toxic effects on the thyroid, influence thyroid function, and damage the thyroid tissue structure (Melzer et al., 2010; Benedetti et al., 2017; Wetherill et al., 2007). Few studies investigated the effects of NP exposure on the morphology and ultrastructure of thyroid tissue. In particular, no relative investigation on the structure of thyroid tissues in neonatal rats after exposure in pregnancy and lactation period has been published. In the present study, we investigated the dose-dependent effect of NP exposed in dams during the perinatal period on the basic growth, development and function as well as structure of the thyroid gland in F1 male rats. This study explored whether exposure to NP at the perinatal period in dams would induce thyroid dysfunction and thyroid tissue damage in F1 male rats by measuring the serum thyroid hormone level, NP levels in thyroid tissue and by observing both the morphology and ultrastructure changes in F1 male rats. To our knowledge, this is the first study to investigate the effects of NP exposed in dams during the perinatal period on thyroid function and structure in F1 male rats. This study can provide research basis for the evaluation of the damage of NP on thyroid tissue structure.

## Materials and Methods

### Vertebrate animal study

Animal experimental procedures were approved by Zunyi Medical University Ethics Committee (2018-1-094). All experiments were performed in accordance with guidelines and regulations of the Zunyi Medical University.

### Treatment and methods

A total of 60 female rats with body weights of 240–260 g and 20 male rats with 290–310 g aged 8 weeks were selected and maintained at 22 ± 2 °C and humidity 50% ± 10%. Food and water were freely available.

**Figure 1:**
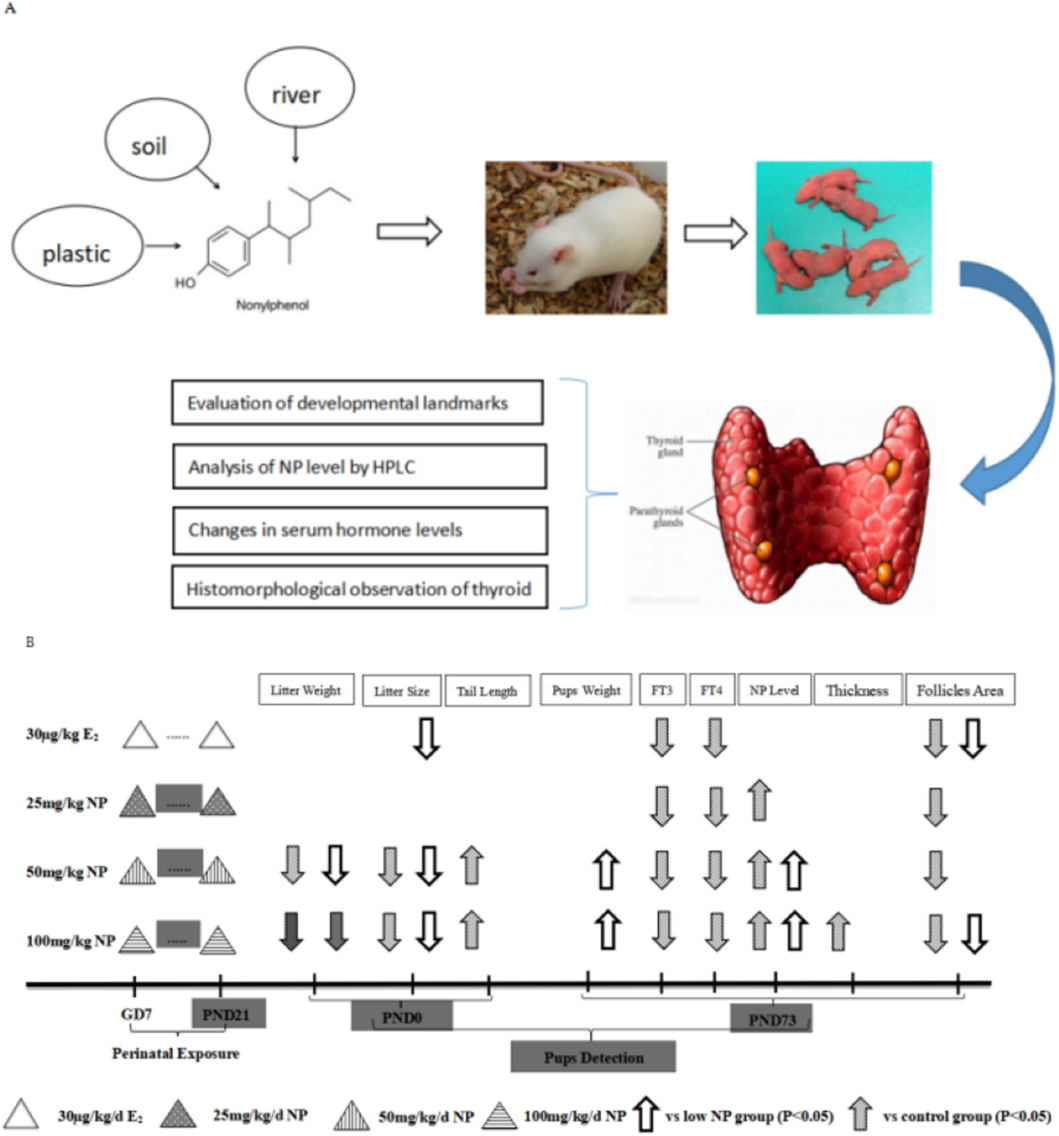
Flowchart depicting exposure protocols and time duration of exposure. (A) Research flowchart; (B) Concrete exposure flowchart.

No rat died after 1-week of acclimation. Female rats were mated with the males at a ratio of 3:1 (four rats per cage). Vaginal secretion was taken at 7 am every day, prepared into smear, and observed by microscope. Pregnancy was successful if sperm was observed, and the day was recorded as Day 0. Then, dams were raised alone. After a successful pregnancy, the rats were randomly grouped. Body weight was recorded every day. NP was orally administrated to the dams based on their body weight after 7 days of pregnancy. Corn oil was given to the control group, and estradiol (E_2_) was given to the positive control group. The rats that received NP were divided into low, middle, and high-dose groups (25, 50, 100 mg/kg/day, respectively). The exposure dose of E_2_ was 30 μg/kg/day, and the volume for gavage was five mL/kg/day. The doses (25, 50, 100 mg/kg/day) were chosen according to our prior study (Xu et al., 2010), which demonstrated that the no observed adverse effect level (NOAEL) of NP was 20–40 mg/kg, therefore, 25 mg/kg was chosen as NOAEL in this study. Moreover, observed adverse effect level of NP was 100 mg/kg, hence 50 mg/kg was chosen according to geometric proportion. The dams were continuously exposed from gestational days 7 until postnatal day (PND) 21 after the neonatal F1 rats were born (ablactation). Only F1 male rats were chosen to assess the toxicity of NP (Xu et al., 2010). Additionally, the dose by factor method, which was described in detail in previous study (Anroop & Shery, 2016), is used to evaluate actual human exposure. The dose by factor method is an empirical approach and use the NOAEL of drug from preclinical toxicological studies to estimate human equivalent dose. A flowchart depicting exposure protocols for the rats and time duration of exposure is shown in Fig. 1.

### Evaluation of developmental landmarks

The rat’s body weight was measured every day, and the dams were given corresponding treatment doses according to their body weight. The times of ear-spreading, hair-germinating, teething, and eye-opening were recorded. Little size and litter weights were measured of different dose groups at birth, and the number of live male pups per little and tail length in each group was recorded.

### Detection of serum thyroid hormones

The F1 male rats were weaned after 21 days of birth and sacrificed after 73 days. Before sacrifice, pups were fasted overnight, and anesthetized by intraperitoneal injection with 20% ethyl carbamate. Blood samples were drawn from the abdominal aorta using an additive-free blood collection tube. The blood samples were centrifuged at approximately 8,000 rpm and 4 °C for 8 min and stored below −80 °C until hormone assay. Serum thyroid hormones free triiodothyronine (FT3), free thyroxine (FT4), and thyroid stimulating hormone (TSH) levels were detected by automated chemiluminescence immunoassay analyzer (ADVID Centaur XP; Siemens Healthcare Diagnostics Inc, Shanghai, China). Detection sensitivities for FT3, FT4, and TSH are 0.2 pg/mL (0.3 pmol/L), 0.1 ng/dL (1.3 pmol/L), 0.004 μIU/mL (mIU/L), respectively, according to the specification of the analyzer.

### Histomorphological observation of the thyroid

After anesthesia, the skin of F1 male rats was incised to expose trachea and to strip the thyroid. The left lobe of the thyroid tissues was immersed in 4% paraformaldehyde, embedded in paraffin, dehydrated, stained with hematoxylin and eosin (HE), prepared into sections, and observed by optical microscope. A subset of the thyroid tissues were immersed in glutaraldehyde fixative and observed by Transmission electron microscopy (TEM).

### HE staining

The thyroids were fixed overnight at 4 °C in 4% paraformaldehyde (pH 7.2), dehydrated through an ethanol series, cleared in xylene, embedded in paraffin, and at least four to five consecutive four-μm sections were cut per sample. Slides were stained with HE according to manufacturer instructions. Image J software was used to quantitatively analyze the HE sections, and measure the thickness of the follicle epithelium and the area of single follicle.

### Transmission electron microscopy

The thyroid glands were fixed in buffered 2.5% glutaraldehyde and 1% osmium tetroxide, dehydrated through an ethanol series, and embedded in epoxy resin. Ultrathin slices of thyroid about 60–80 nm were made by ultramicrotome. The ultra-structures of follicular epithelial cells were examined with a transmission electron microscope (HT7700; Hitachi, Tokyo, Japan).

### Analysis of NP level by HPLC

The right lobe of thyroid tissues was stored by dry preservation for NP level detection by high-performance liquid chromatography (HPLC) as described by Luo et al. (2015). Briefly, 10 mg of minced samples of thyroid were mixed with hexane/diethylether, homogenized, and then centrifuged. The supernatant was evaporated in a water bath. HPLC analysis was performed using a HP-1100 system (Agilent, Santa Clara, CA, USA). An Eclipse XD8-C18 column (Agilent, Santa Clara, CA, USA) was used for the separation. The mobile phase was composed of acetonitrile (eluent A) and 0.1% glacial acetic acid (eluent B) (A/B = 85/15, v/v). The injection volume was 10 μL. The excitation and emission wavelengths were 275 and 312 nm, respectively (Luo et al., 2015).

### Statistical analysis

SPSS software, version 20.0 for Windows (SPSS Inc., Chicago, IL, USA) was used to analyze the data. Values of all variables are presented as means ± SD. One-way analysis of variance was applied to analyze the data, and the least significant difference (LSD) procedure was used to test differences between groups. Statistical significance was assumed at *P* < 0.05. Image J was used to quantitative analyze and measure the thickness of the follicle epithelium and the area of single follicles.

## Results

### Changes of developmental landmarks

**Figure 2:**
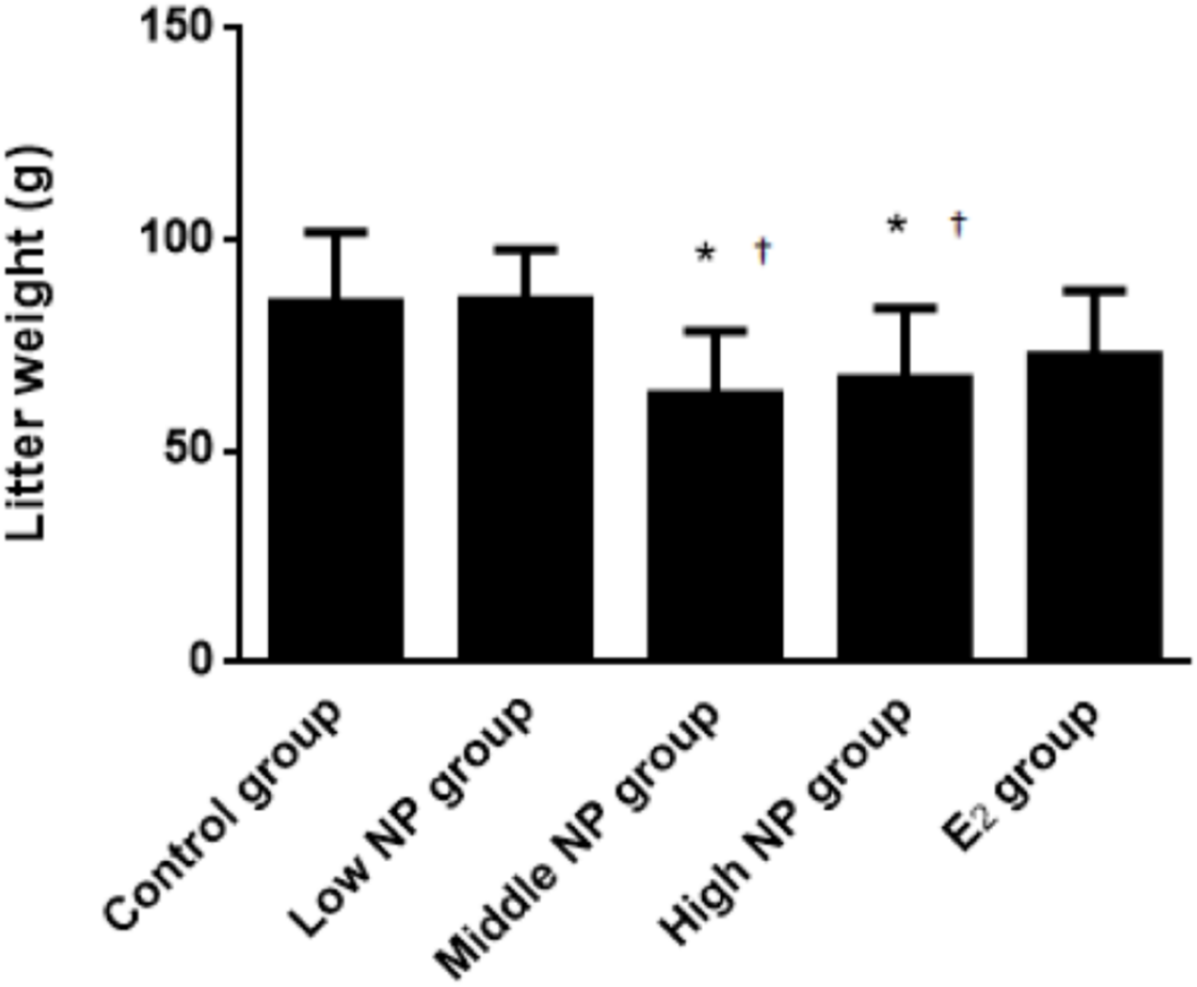
Comparison of the litter weight of male pups on PND 0 among different treatment groups. *n* = 6–9, *vs control, *P* < 0.05; †vs low NP group, *P* < 0.05.

**Figure 3:**
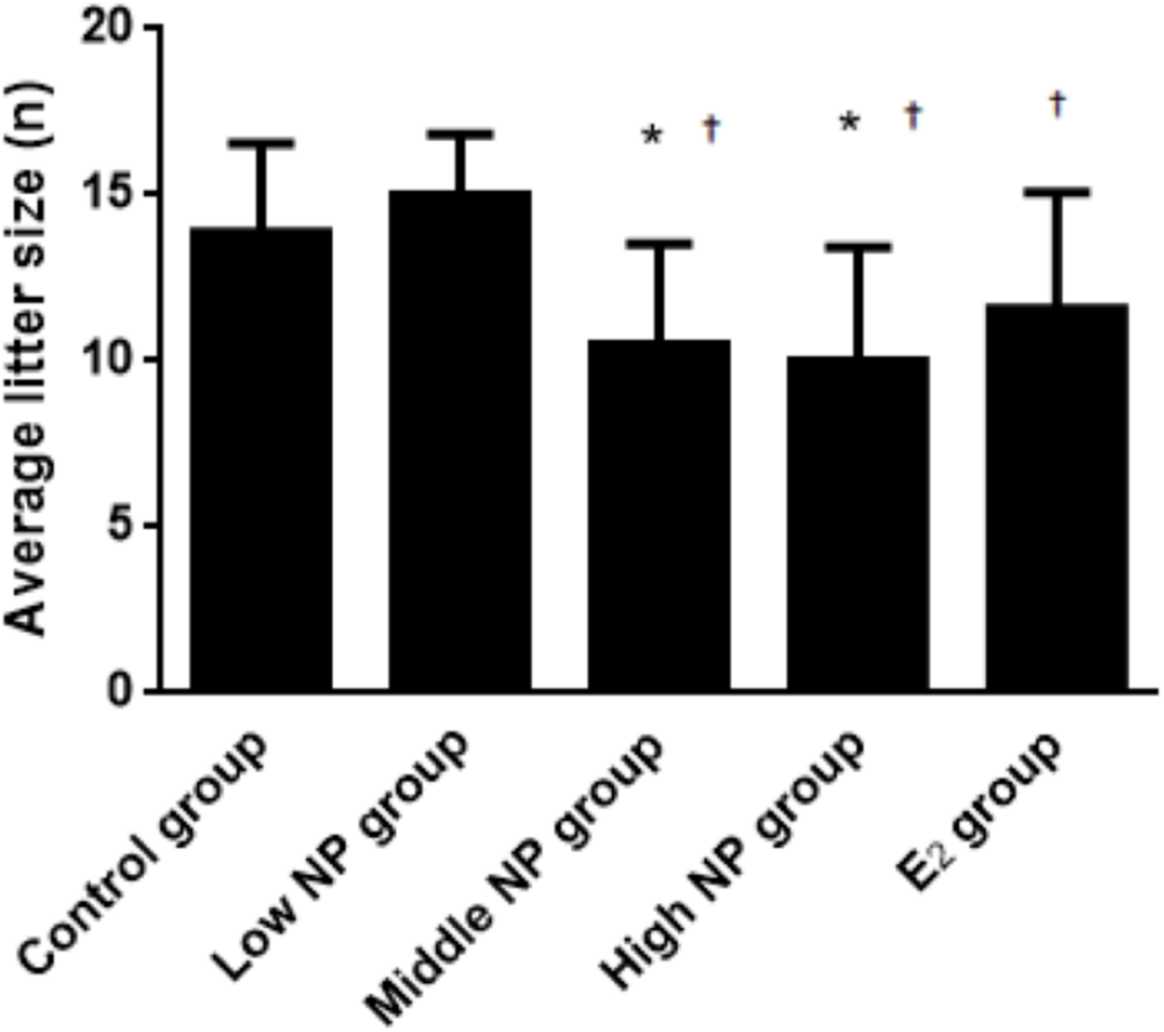
Comparison of average litter size on PND 0 among different treatment groups. *n* = 7–10, *vs control, *P* < 0.05, †vs low NP group, *P* < 0.05.

**Figure 4:**
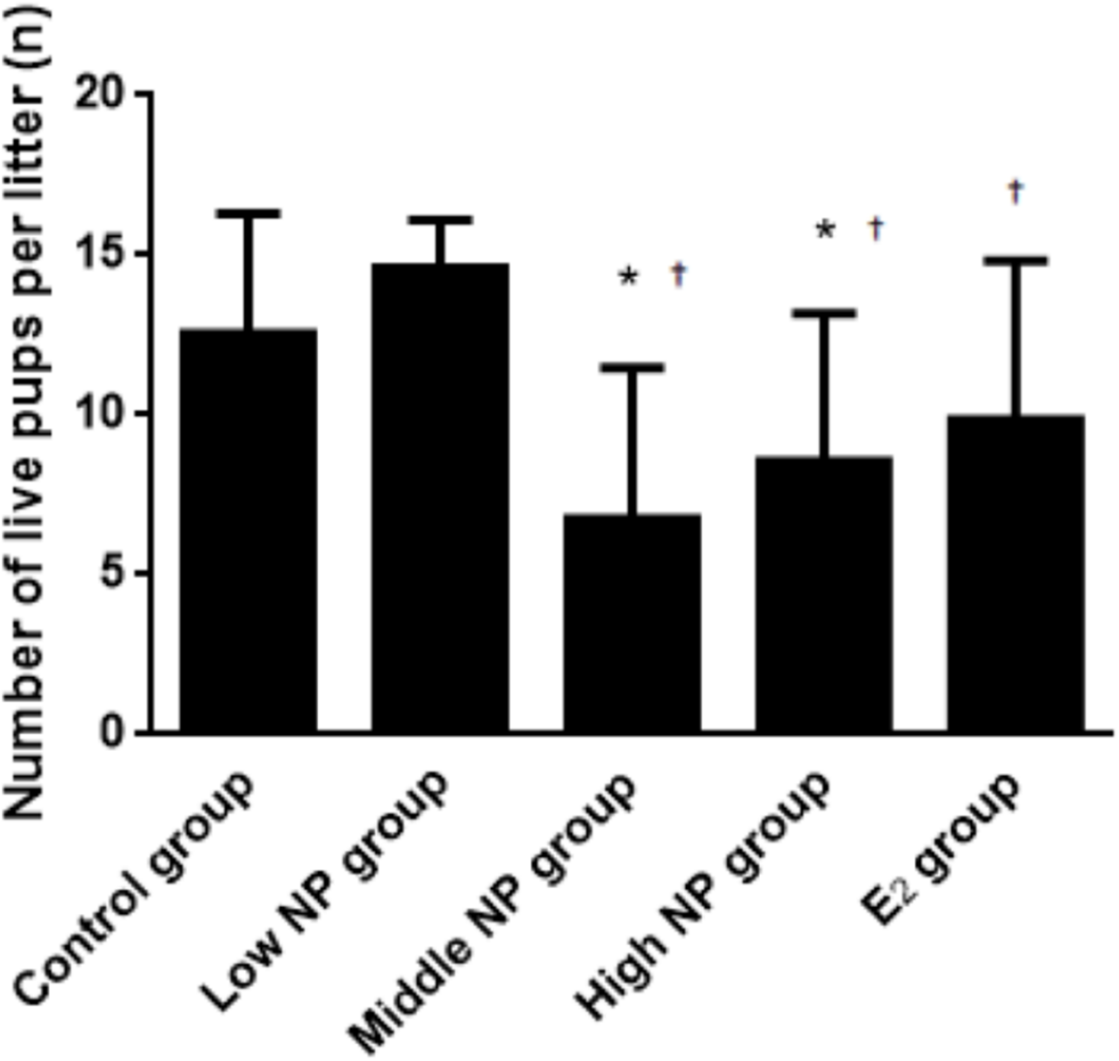
Comparison of the number of live pups per litter on PND 0 among different treatment groups. *n* = 7–10, *vs control, *P* < 0.05, †vs low NP group, *P* < 0.05.

**Figure 5:**
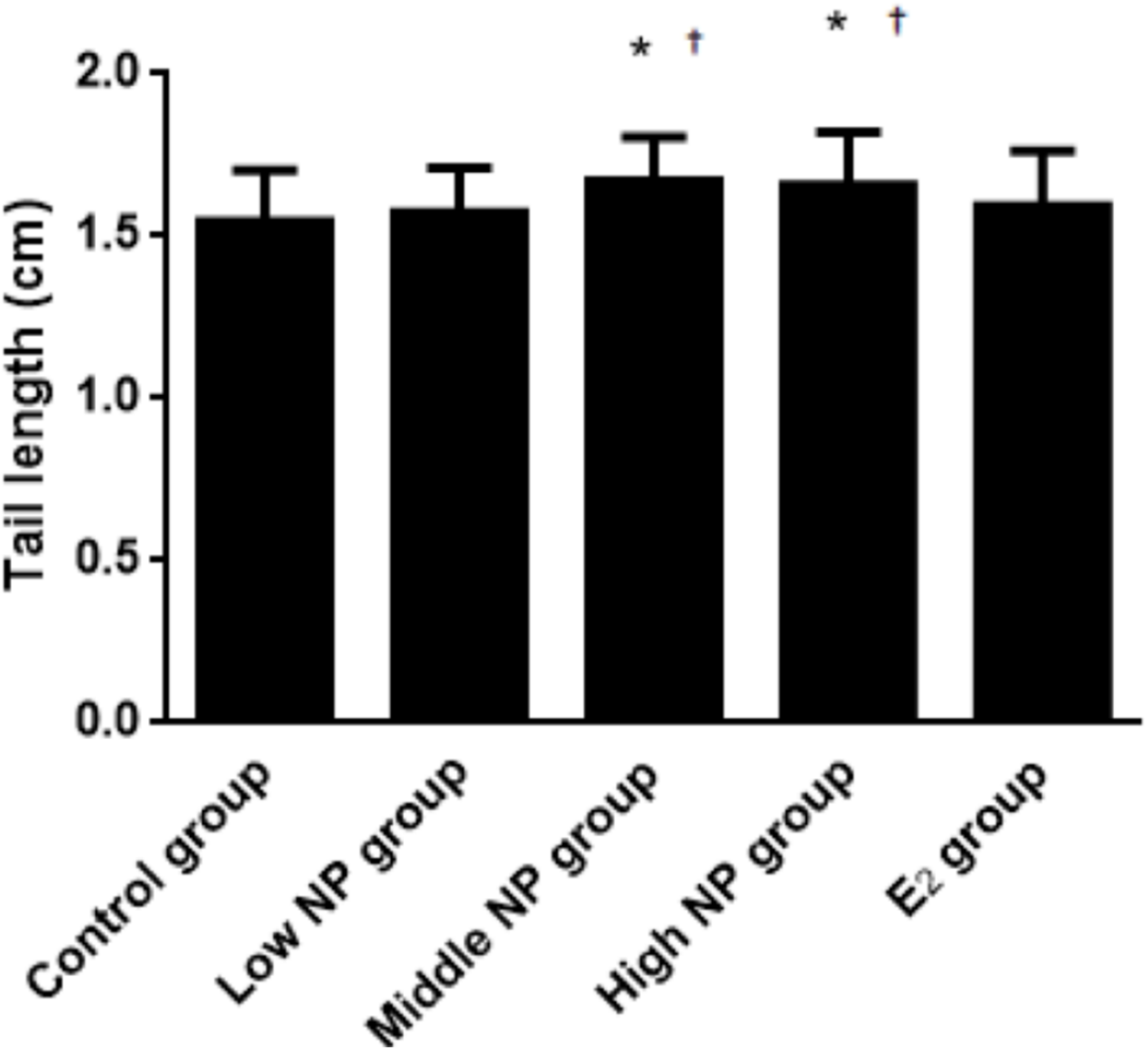
Comparison of tail length among male pups on ND 0 among different treatment groups. *n* = 17–43, *vs control, *P* < 0.05; †vs low NP group, *P* < 0.05.

On PND 0, exposure to 50 and 100 mg/kg/day NP caused a significant decrease in litter weight in comparison to the control and low NP groups on PND 0 (*P* < 0.05, Fig. 2). Oral exposure to NP showed a statistically significant effect on the average litter size and number of live pups per litter compared to the control group (*P* < 0.05). Exposure to 50 and 100 mg/kg/day NP produced a significant decrease in the average litter size and number of live pups per litter on PND 0 (*P* < 0.05, Figs. 3 and 4). Additionally, the tail length of pups increased in the 50 and 100 mg/kg NP groups compared to the control group (*P* < 0.05, Fig. 5). No significant difference in the body weight of pups among treatment groups was found.

**Figure 6:**
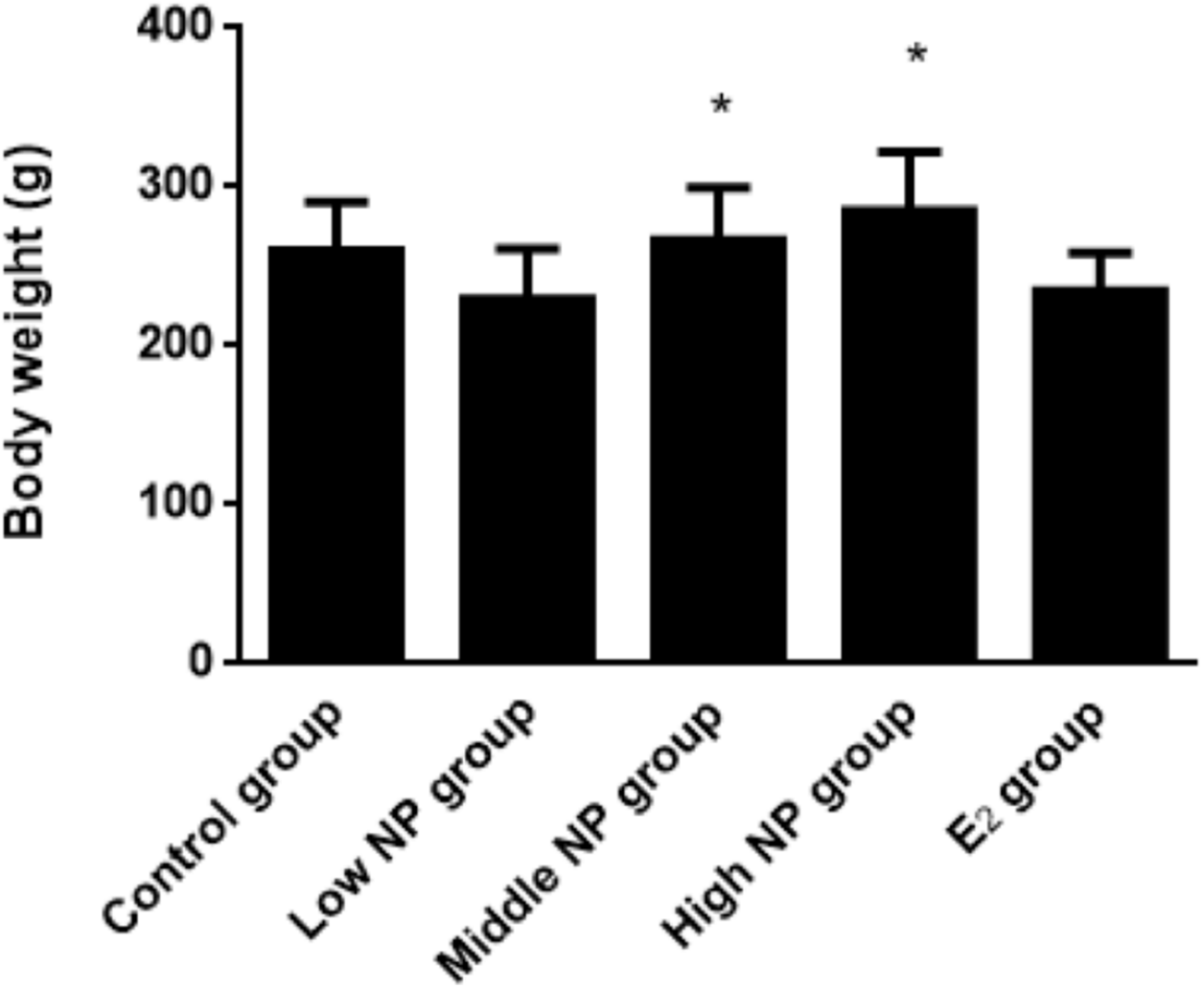
Comparison of body weight of male pups on PND 73 among different treatment groups. *n* = 8–10, *vs low NP group, *P* < 0.05.

On PND 73, a significant difference in body weight was found among pups. Among the NP treatment groups, NP affected the body weight in the pups in a dose-dependent manner (*P* < 0.05), but no significant difference was found between the NP treatment groups and the control group (Fig. 6). In addition, NP had no effect on the developmental landmark time (ear-spreading, hair-germinating, teething, and eye-opening) in male pups from PND 0 to PND 15.

### Changes of serum thyroid hormone levels

**Figure 7:**
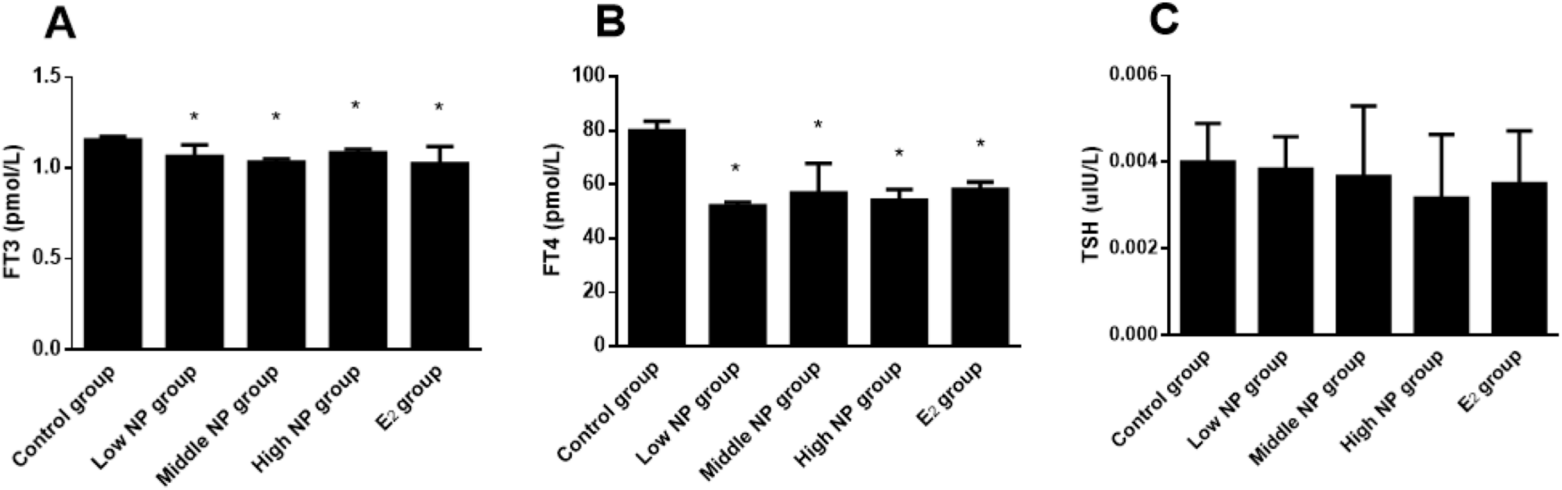
Comparison of serum FT3, FT4, and TSH levels in F1 male rats on PND 73 among different treatment groups. *n* = 6, *vs control, *P* < 0.05.

Dams exposed to NP during perinatal period demonstrated decreased serum levels of FT3, FT4, and TSH in F1 male rats, when compared to the control group. Significant differences in serum FT3 (*P* < 0.05) and FT4 (*P* < 0.05) levels rather than serum TSH level were found among treatment groups (Fig. 7).

### NP level in the thyroid

**Figure 8:**
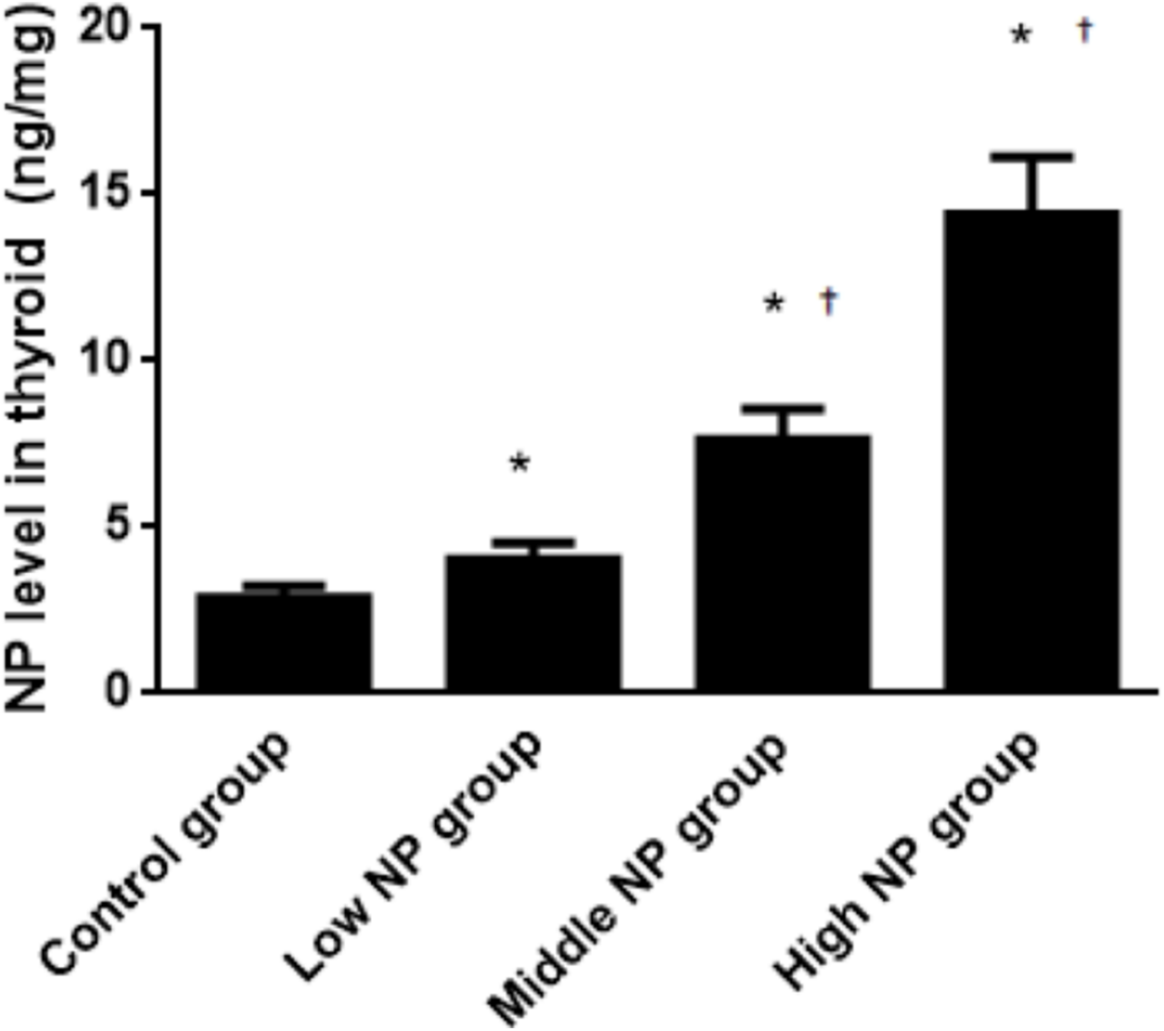
Comparison of the NP level in the thyroids of pups on PND 73 among different treatment groups. *n* = 10, *vs control, *P* < 0.01; †vs low NP group, *P* < 0.01.

The NP level of the control group was 3.39 ± 0.08 ng/mg, while NP levels of the low, middle, and high-dose groups were 5.45 ± 0.19, 6.78 ± 1.09, and 9.90 ± 0.60 ng/mg, respectively. NP levels in the low, middle, and high-dose groups were significantly higher than in the control group (*P* < 0.01). Similarly, the NP levels were significantly higher in the high and middle-dose groups compared to the low dose group (*P* < 0.01). Exposure caused a dose-related increase in NP level in the thyroids of male pups (*P* < 0.01, Fig. 8).

### Histological change of the thyroid

#### Morphological change of the thyroid

**Figure 9:**
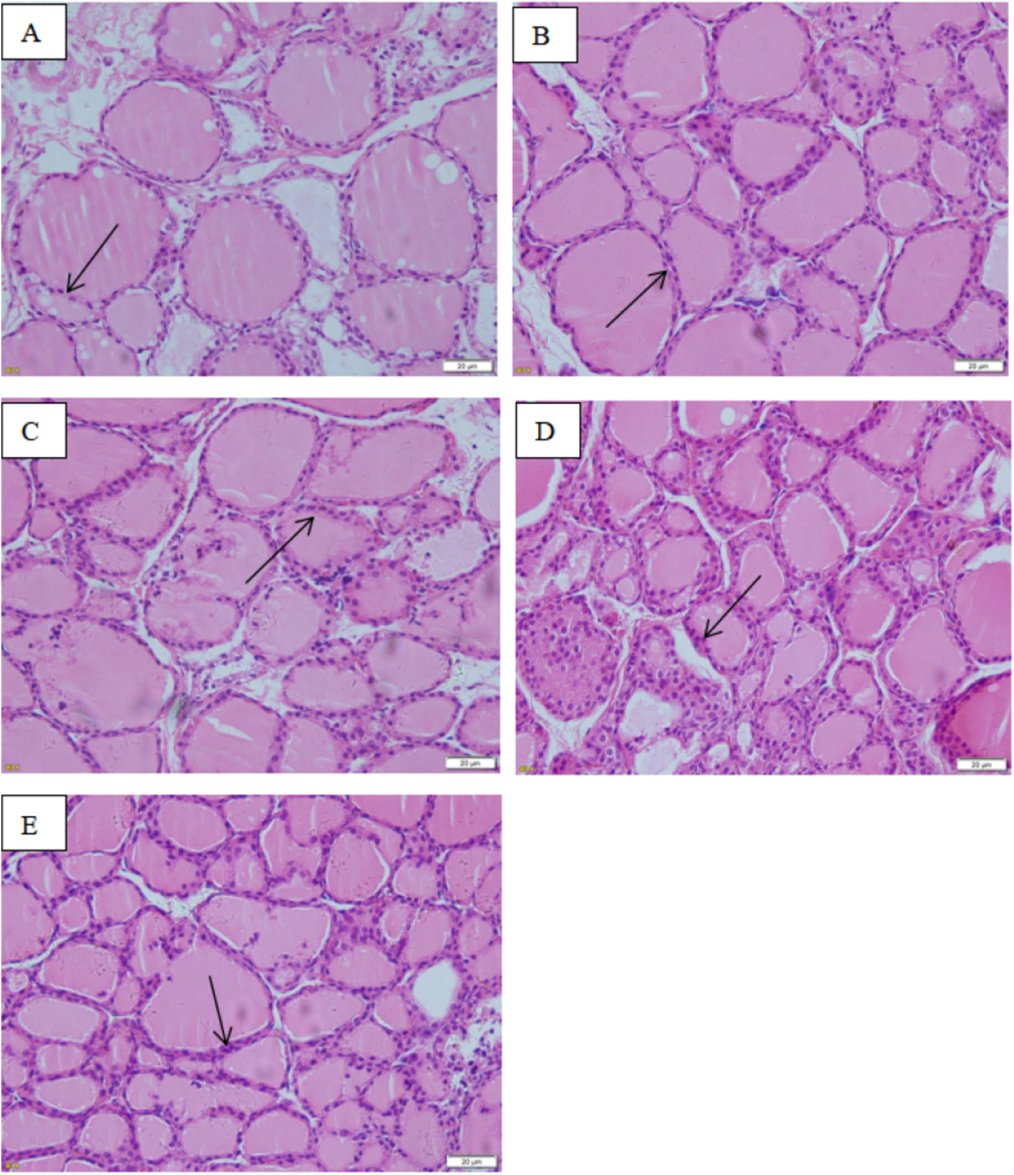
Effect of maternal NP exposure on thyroid morphology in pups. Magnification 400×. (A) control group, (B) low-dose group, (C) middle-dose group, (D) high-dose group, (E) estradiol (E2) group. Arrow: thyroid follicular epithelium.

The images taken with the optical microscope illustrate that the thyroid follicle epithelium in the control group is simple cuboidal, and many large thyroid follicles could be observed, which are filled with red jelly (Fig. 9). A small quantity of stratification was observed in the follicle epithelium in the low-dose group, and a large quantity was observed in the middle-dose, high-dose and positive control groups.

**Figure 10:**
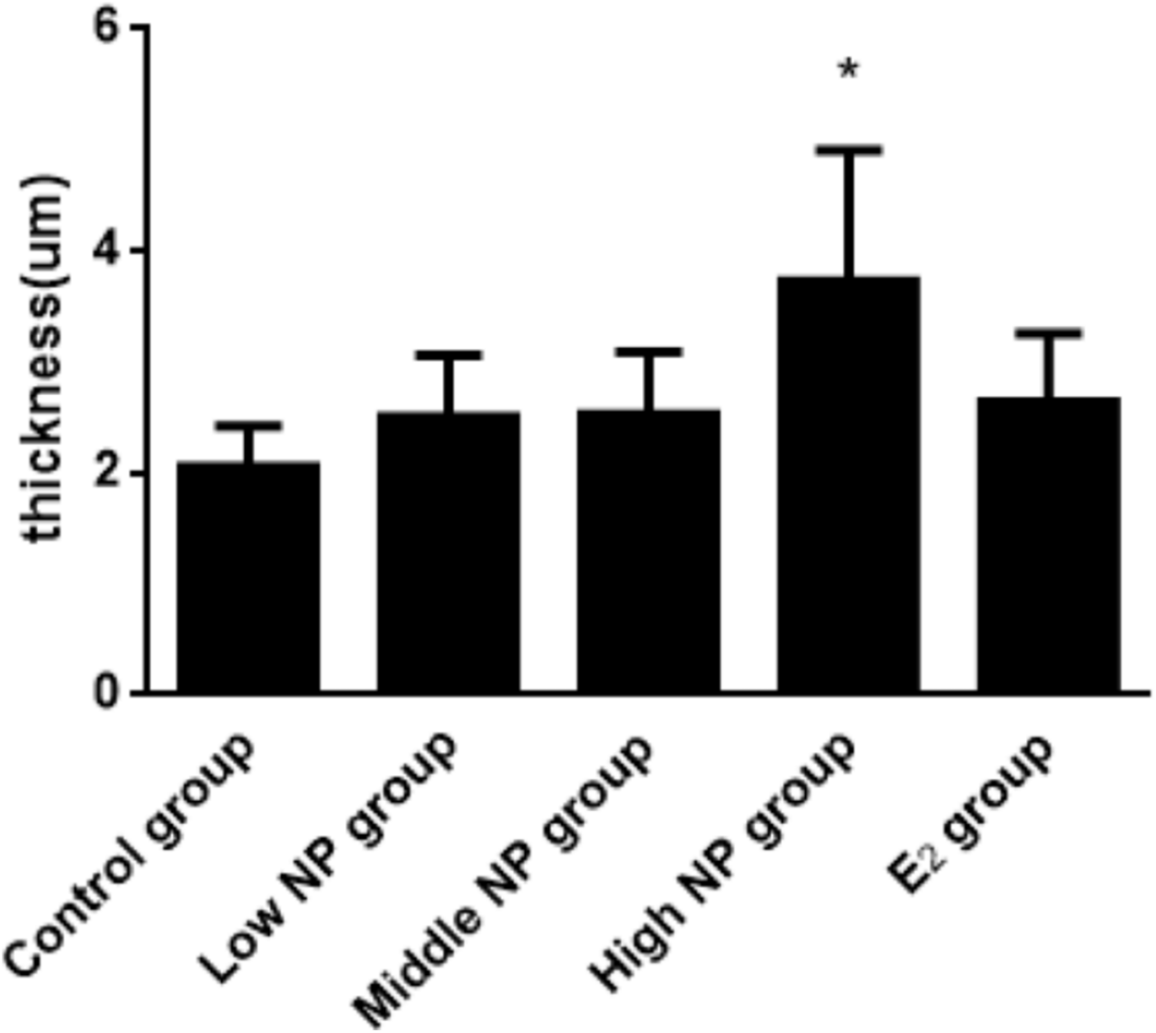
Comparison of the thickness of thyroid follicular epithelia among different treatment. *vs control, *P* < 0.05.

**Figure 11:**
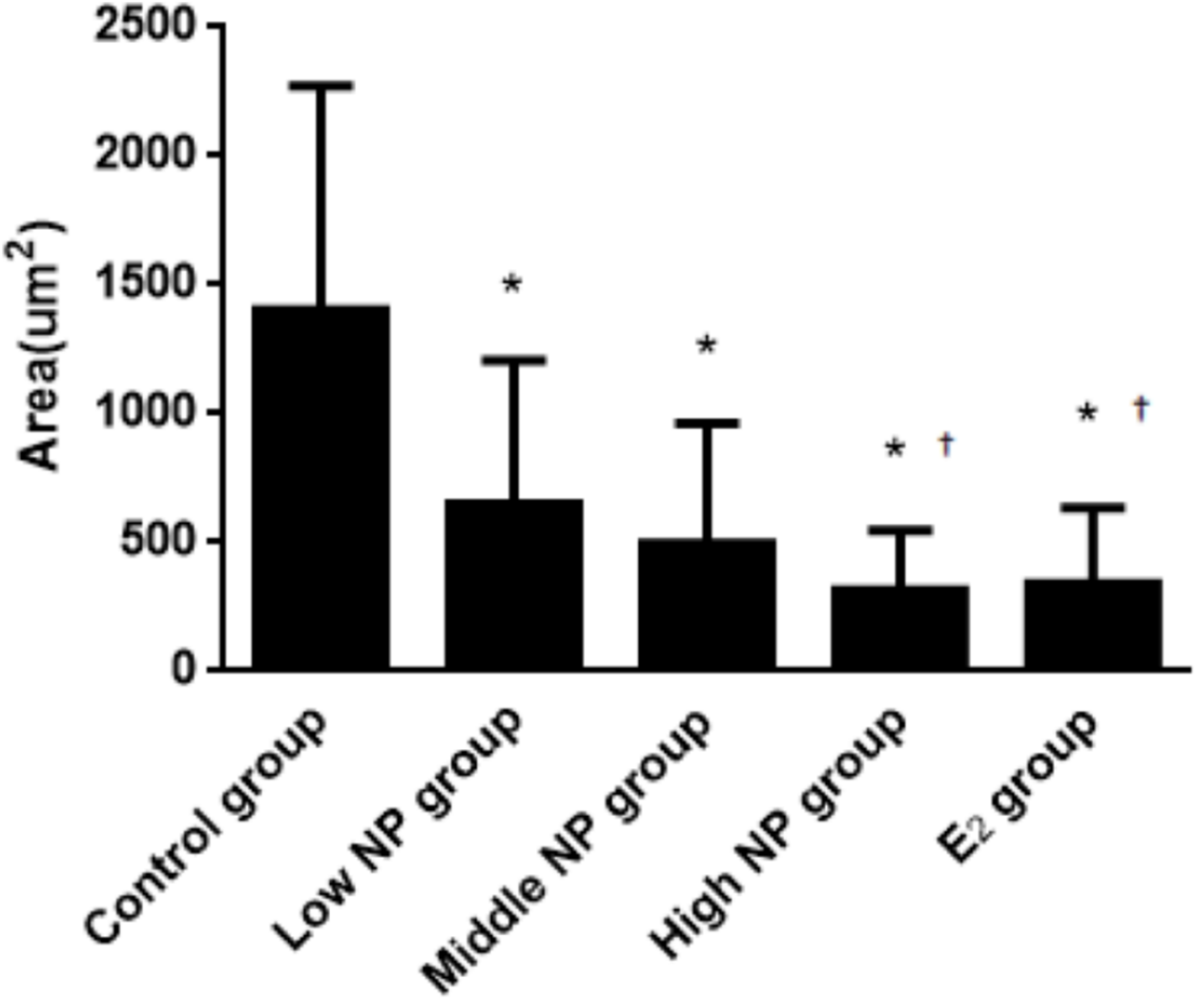
Comparison of the size of thyroid follicles among different treatment groups. *vs control, *P* < 0.01; †vs low NP group, *P* < 0.05.

The thicknesses of the thyroid follicular epithelium in the control, low, middle, high-dose, and E_2_ groups were 2.06 ± 0.37, 2.51 ± 0.56, 2.53 ± 0.56, 3.97 ± 1.61, and 2.64 ± 0.62 μm, respectively. The thickness of the thyroid follicular epithelium increased with an increase in treatment dose in a dose-dependent manner (*P* < 0.05, Fig. 10). The sizes of the thyroid follicle in the control, low, middle, high-dose, and E_2_ groups were 1,405.53 ± 866.62, 651.04 ± 555.69, 496.38 ± 467.34, 317.49 ± 231.15, and 341.84 ± 293.82 μm^2^, respectively. With an increase in NP treatment dose, animals showed a decrease of the size of thyroid follicles in the thyroid (*P* < 0.01, Fig. 11).

#### Ultrastructure changes of the thyroid

**Figure 12:**
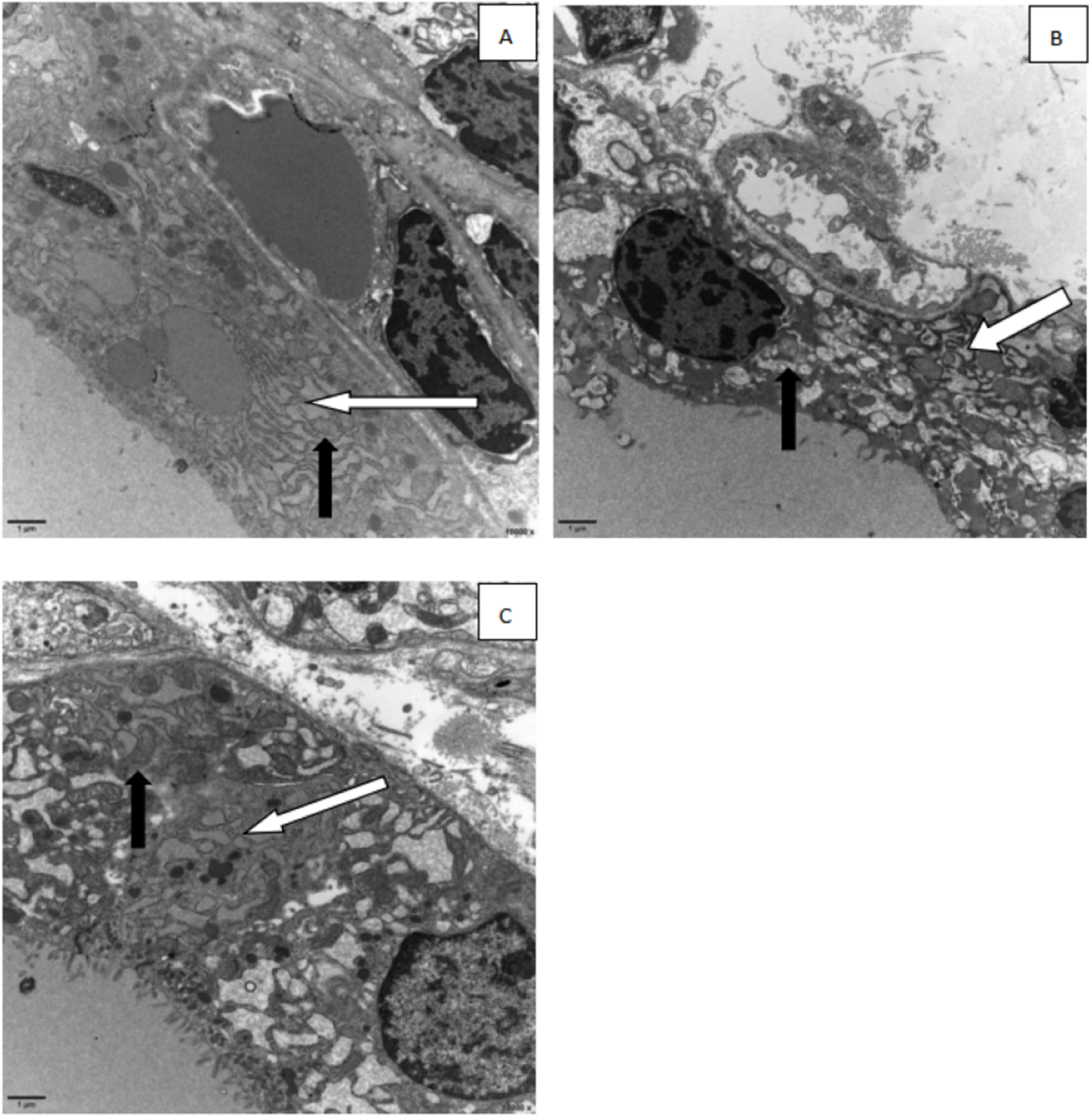
Electron micrographs of thyroid follicular cells from different treatment groups. Black arrow: mitochondria, white arrow: endoplasmic reticulum). (A) control group; (B) 100 mg/kg NP group; (C) estradiol (E2) group.

The cellular morphology in the control group was normal. No swelling was found in mitochondria, and no expansion was found in the rough endoplasmic reticulum. Several cell nuclei were quasi-circular and irregular, dominated by euchromatin. In the thyroid follicle epithelial cells in the NP groups, mitochondria were swollen to a certain degree. The rough endoplasmic reticulum showed expansion with irregular vesicles. The shapes of cell nuclei were quasi-circular, and more chromatin condensation was found in the control group. The expansion degree of the rough endoplasmic reticulum in the positive control group was more severe. The small vesicles fused into irregular large vesicles. The swelling degree of mitochondria became more severe, and the cell nuclei represented irregular quasi-circular shape, dominated by euchromatin, with appearance of condensation of part chromatin (Fig. 12).

## Discussion

To our knowledge, this is the first study that reports the NP level in the thyroid by HPLC, and examined the negative effects on the thyroid structure in male F1 rats after gestational and lactational exposure of dams to NP. The main findings of our study are that NP impaired well-established diagnostic markers of growth such as the average litter size, litter weight of pups, number of live pups per litter, tail length, body weight of pups, serum thyroid hormone level, and thyroid ultrastructure. NP exposure also caused alterations in the thyroid follicular epithelium, the mitochondria, and the endoplasmic reticulum in the thyroid follicular epithelial cells. In addition, exposure caused a dose-dependent increase in the NP level in the thyroid glands of male pups.

Nonylphenol widely exists in rivers, soil, and food, and possesses many characteristics such as difficulty of degradation, long-term residual, and bioaccumulation. It can accumulate in animals or humans via the food chain (Crane, 2019; Günther, Räcker & Böhme, 2017). The thyroid is an important endocrine organ, and NP exposure has been reported to interfere with its function (He et al., 2018; Naderi et al., 2014), leading to endocrine disorder and influencing both basic growth and development. Most of the current investigations on the effect of NP on thyroid function mainly focus on short-term exposure using serology detection. No study has used HPLC for the detection of the NP levels in thyroid tissues, nor on the thyroid tissue injury of neonatal rats after maternal rats were exposed during the perinatal period. In this study, the maternal perinatal period was used for NP exposure and the resulting influences of NP on the basic growth and development as well as both the structure and ultrastructure of thyroid tissue were investigated.

After dams were exposed to NP, no difference was found in body weight between groups, and their weight growth was normal, suggesting that NP might not interfere with the body weight growth of pregnant rats at the tested doses or the survival rate of neonatal rats. Even so, the numbers of neonatal rats in the medium and high dose groups were decreased, and the tail length was increased significantly compared to the control group. The results of neonatal rats indicate that no statistical difference was observed in birth weight, anogenital distance, times of ear-spreading, hair-germinating, teething, and eye-opening at the early stage of growth and development, which indicates that the weight in the high dose group was significantly higher than in the low dose group. This suggests that high NP exposure during the perinatal period could intervene with the basic growth and development of offspring, which has not been investigated in before. Because exposure to NP did not stop during the lactation period, NP could be detected in the milk at a certain level (Xie, Liu & Chen, 2014). The NP may have entered the neonatal rats via milk, and further affected growth and development of neonatal rats. Thus, it can be confirmed that exposure to NP during the perinatal period influences the basic growth and development of neonatal rats in a dose-dependent manner.

Thyroid hormones are necessary for growth, development and normal bodily function. Alterations in thyroid hormone level are one of the important indicators when thyroid function changes in the body. In the present study we found that dams exposed to NP during perinatal period demonstrated decreased serum levels of FT3, FT4 in F1 male rats, which was consistent with previous study on the thyroid toxicity of endocrine disruptor (Serrano-Nascimento et al., 2018).These findings show that NP could lead to the hypoactivity of the thyroid gland.

With increasing exposure dose, the NP level in thyroid tissues increased continuously, and there was a statistical difference between each experimental group and the control group (*P* < 0.05). The high dose group had a statistical difference between the control group, the low-dose group, and the medium-dose group (*P* < 0.05). This shows that NP can be accumulated in the body and transmitted into offspring. Other EEDs, such as BPA and PCBs, have been widely investigated. It has been reported that after female pregnant rats were exposed to BPA at a certain dose, the thyroid function of neonatal rats was disordered, and the tissue will be injured after being exposed to PCBs at a certain level (Tang et al., 2013). Although there are many relevant investigations, the detection of NP levels in the thyroid has not been reported. Through measuring the NP level in tissue, the thyroid tissue damage could be confirmed to be directly caused by the NP in tissues.

This result indicates that thyroid tissues in the control group are relatively normal, and the change degree in each exposure group was different. With increasing exposure dose, the stratification in thyroid follicle epithelium becomes more severe. The number of small follicles increased, and colloid area in the follicle decreased. According to the statistics, the thickness of the follicle epithelium shows a statistical difference between the high NP group and control group (*P* < 0.05). Although no significant difference was found between each group and the control group, the thickness increased slightly. The result of the thyroid follicle area demonstrates a statistical difference between each group and the control group (*P* < 0.05). The area of follicle continuously decreased with increasing exposure dose. These results are similar to the results of Xi, Li & San (2013), because exposure time, dose, and subjects are different, as well as the detected objects after exposure, the injury degree on tissue were also different. However, it has been confirmed that the exposure in the perinatal period damages the structure of thyroid tissue.

It has been reported that the function of the thyroid is actively impaired by external factors (Xi, Li & San, 2013; Andrade et al., 2018). The most significant manifestation is a morphologic change of thyroid follicular cells. For example, the thickness of the epithelium increased and the number of small follicles increased, while the area of follicles decreased. NP exposure of pregnant rats damages the thyroid tissues of offspring. It is highly possible that long-term severe thyroid damage will further aggravate the hyperplasia of the thyroid, or even lead to a thyroid nodule. Under TEM, the organelles in the control group were normal without significant damage. Moreover, mitochondria and rough endoplasmic reticulum were also normal. Several of the cell nuclei showed quasi-circular and slightly irregular shapes, dominated by euchromatin in the cell nucleus. The NP exposure group showed that mitochondria were swollen to a certain degree. The rough endoplasmic reticulum showed expansion with vesiculation. The shape of the cell nucleus was quasi-circular, and more chromatin condensation was observed in the control group. The expansion degree of the rough endoplasmic reticulum in the positive control group was more severe, as was vesiculation. Small vesicles fused into irregular large vesicles. The swelling degree of mitochondria became more severe, and the cell nucleus showed an irregular quasi-circular shape, dominated by euchromatin with appearance of chromatin condensation.

It has been reported that mitochondrial swelling is mainly caused by deficient production of cell energy under hypoxia (Onukwufor, Stevens & Kamunde, 2016; Wu, 2003). Under physiological stress, mitochondrial function increased, and mitochondria increased slightly. However, the result of TEM images indicates that the mitochondrial matrix was swollen in thyroid cells in both the NP group and the estradiol control group. Thus, exposure to NP leads to cell damage. For the endoplasmic reticulum, expansion of the endoplasmic reticulum has been reported (Wu, 2003) and the appearance of vesiculation suggests a reduction of cell function. In general, this is caused by various distortions or necrotic lesions. The TEM images in the NP exposure and positive control groups also illustrates expansion and vesiculation. Thus, exposure to NP will damage thyroid cells.

Investigating sexual dimorphism is important for our understanding of its influence on thyroid function and structure. One limitation of the present study needs to be acknowledged. Only F1 male rats were chosen for detecting the toxicity of NP. The reason was that the thyroid hormone will fluctuate with the menstrual cycle, which is to the disadvantage of detection. In addition, F1 male rats were chosen according to our prior study (Yu et al., 2013; Xu et al., 2010), which showed that the estrogen-like effect of NP on reproductive and nerve systems was mainly tested in male rats.

## Conclusion

Although the mechanism of NP exposure on the structural change of thyroid tissue, as well as the microstructural changes of mitochondria and endoplasmic reticulum still remain unclear, we found that oral NP exposure of dams during pregnancy and lactation influence the basic growth and development of neonatal rats. NP exposure of maternal rats will enter into neonatal rats and accumulates in the thyroid tissue, directly damaging the structure of the thyroid tissue and affecting the normal function of the thyroid.

## Supplemental Information

10.7717/peerj.7039/supp-1 Supplemental Information 1 Raw data used for data analyses and preparation for Influence of nonylphenol exposure on basic growth, development, and thyroid tissue structure in F1 male rats.

## Additional Information and Declarations

### Data Availability

The following information was supplied regarding data availability: Raw data is available as a Supplemental File.
